# External Use of Propolis for Oral, Skin, and Genital Diseases: A Systematic Review and Meta-Analysis

**DOI:** 10.1155/2017/8025752

**Published:** 2017-02-06

**Authors:** Soo-Hyun Sung, Gwang-Ho Choi, Nam-Woo Lee, Byung-Cheul Shin

**Affiliations:** ^1^Department of Policy Development, National Development Institute of Korean Medicine, Seoul 04554, Republic of Korea; ^2^School of Korean Medicine, Pusan National University, Yangsan 50612, Republic of Korea; ^3^Division of Clinical Medicine, School of Korean Medicine, Pusan National University, Yangsan 50612, Republic of Korea

## Abstract

*Objective.* The aim of this review is to provide the available evidence on the external use of propolis (EUP) for oral, skin, and genital diseases.* Method.* We searched twelve electronic databases for relevant studies up to June 2016. Randomized clinical trials (RCTs) were included and analysed.* Results.* Of the 286 articles identified, twelve potentially relevant studies met our inclusion criteria. A meta-analysis of two studies on recurrent oral aphthae (ROA) indicated that there were no significant differences in total effective rate (TER) for pain disappearance between EUP and placebo groups (RR = 1.96, 95% CI = 0.97–3.98, and *P* = 0.06). In two studies on skin diseases, the combined treatment of EUP with other interventions revealed significant effects on the duration of treatment or TER. In one study on genital diseases, EUP showed significant differences in genital herpes outcome measures compared to placebo.* Conclusions.* Our results on the effectiveness of EUP for treating oral, skin, and genital diseases are not conclusive because of the low methodological qualities and small sample sizes. Further well-designed randomized controlled trials, with high quality and large samples for specific disorders, must be conducted to obtain firm conclusions.

## 1. Introduction

Propolis, also known as “bee glue,” is a wax-like substance that is collected from local flora by honeybees to protect and repair their hives [1, 2]. Humans have been using propolis since ancient times, from at least 300 BC, and there are records suggesting that propolis has been used as a medicine in many parts of the world, both internally and externally [3, 4]. In general, propolis contains phenol acids, flavonoid, terpenes, aromatic aldehydes and alcohols, fatty acids, stilbenes, *β*-steroids, and various other substances [5, 6].

Many researchers have studied propolis in recent decades. The major components extracted from propolis have shown antimicrobial activity [7], and the treatment of cells with ethanolic extract of propolis (EEP) has exhibited anti-inflammatory activity [8]. EEP has also been reported to exhibit antitumour effects in cancer cells [9, 10].

The external use of propolis is defined by the application of pharmaceutical or natural products on the surface or point of illness [11]. External uses of propolis (EUP) include the use of pharmaceutical, cosmetic, and oral products such as ointment [12], gel [13], lipstick [14], and mouthwash [15].

A recently published systematic review on propolis for oral health reported that it can reduce oral infection and dental plaque and treat stomatitis [16]. However, no published studies to date have evaluated the effectiveness of propolis for external use. In addition, numerous published randomized controlled trials (RCTs) on Complementary and Alternative Medicine (CAM) in Korea could be omitted if the database searches are restricted to English- and Chinese-language databases [17]. Korean CAM RCTs are typically missed in systematic reviews, which can increase the risk of language bias [17].

Therefore, we conducted a systematic review and meta-analysis following the PRISMA recommendations [18]. The aim of this systematic review is to explore the evidence on the effectiveness of the external use of propolis for oral, skin, and genital diseases.

## 2. Methods

### 2.1. Data Sources and Searches

We searched the following electronic databases up to June 2016 without a language restriction: MEDLINE (OvidSP), EMBASE (OvidSP), the Cochrane Central Register of Controlled Trials (CENTRAL), and CINAHL Plus (EBSCOhost). We also searched six Korean medical databases (Korea Institute of Science and Technology Information, Korean traditional knowledge portal, KoreaMed, OASIS, RISS, and the National Library of Korea) and two Chinese databases (CNKI and Wanfang). Furthermore, we conducted nonelectronic searches of conference proceedings, our own article files and nine traditional Korean medical journals (Journal of Korean Medicine, the Journal of Korean Acupuncture and Moxibustion Society, Korean Journal of Acupuncture, Journal of Acupuncture and Meridian Studies, Journal of Pharmacopuncture, Journal of Oriental Rehabilitation Medicine, the Journal of Korean Chuna Manual Medicine for Spine and Nerves, Korean Journal of Oriental Physiology and Pathology, and the Journal of Korean Oriental Internal Medicine).

The following search terms were used in each database's language: “propolis” AND “external use OR external application OR external treatment OR topical application OR ointment OR gel OR dressing OR oral OR skin OR genital” AND “randomized controlled trial OR randomized clinical trial”.

### 2.2. Study Selection

#### 2.2.1. Inclusion Criteria

We defined EUP interventions as any type of intervention in which propolis ingredients were applied to illness points as a treatment. All RCTs evaluating EUP for various diseases were included. Patients diagnosed with any disease were also included. We classified each disease according to the International Statistical Classification of Diseases and Related Health Problems, 10th Revision (ICD-10) [31].

Studies on the combined effects of EUP and other interventions (e.g., EUP plus rinsing therapy) were considered for inclusion when the same intervention was applied to both the EUP group and the control group.

Clinical trials comparing EUP with placebo or other active controls were included. Other active control interventions included rinsing therapy, miconazole, oral antiseptics, silver sulfadiazine, honey, Vaseline, pine pollen packs, and metronidazole gel.

#### 2.2.2. Exclusion Criteria

Non-RCTs, animal or cell studies, and quasi-RCTs were excluded. Trials including healthy participants were also excluded. We did not include studies on the internal use of propolis (e.g., propolis capsules, tablets, or suspensions) or mouthwash interventions (e.g., mouth rinsing, teeth brushing). Unqualified control interventions (e.g., herbal medicine, acupuncture, and bee venom therapy) were excluded because their efficacy was unable to be investigated.

### 2.3. Data Extraction

Three authors (S. H. Sung, G. H. Choi, and N. W. Lee) independently selected the included studies and extracted data using a predefined data extraction form. N. W. Lee, who is a Traditional Chinese Medicine (TCM) practitioner, searched the Chinese databases and screened the Chinese-language trials. For studies with insufficient information, we contacted the corresponding authors to request additional data. Disagreements were resolved by discussion between two authors (G. H. Choi and B. C. Shin) to reach consensus.

### 2.4. Assessment of Risk of Bias (ROB)

We used the Cochrane risk of bias tool [32]. This tool includes 7 domains, but we assessed random sequence generation, allocation concealment, blinding of participants or personnel, blinding of assessors, incomplete outcome data, and selective outcome reporting. The risk of bias in each study was assessed by two independent authors (S. H. Sung and G. H. Choi) using the Cochrane risk of bias tool; disagreements were resolved by discussion.

### 2.5. Data Analyses

For meta-analyses, we extracted dichotomous data using risk ratios (RR) for the total effective rate (TER) for pain disappearance. We applied a random-effects model using Review Manager (Revman) software (version 5.3 for windows; the Nordic Cochrane Centre, Copenhagen, Denmark). *I*^2^ tests were used to analyse the heterogeneity between the included studies. *I*^2^ values above 50% were considered to indicate possible heterogeneity [32]. As statistical pooling was not feasible due to the variability of diseases, types of EUP form, control interventions, and outcome measures, a summary of the findings is presented in the results.

## 3. Results

### 3.1. Study Selection and Description

**Figure 1:**
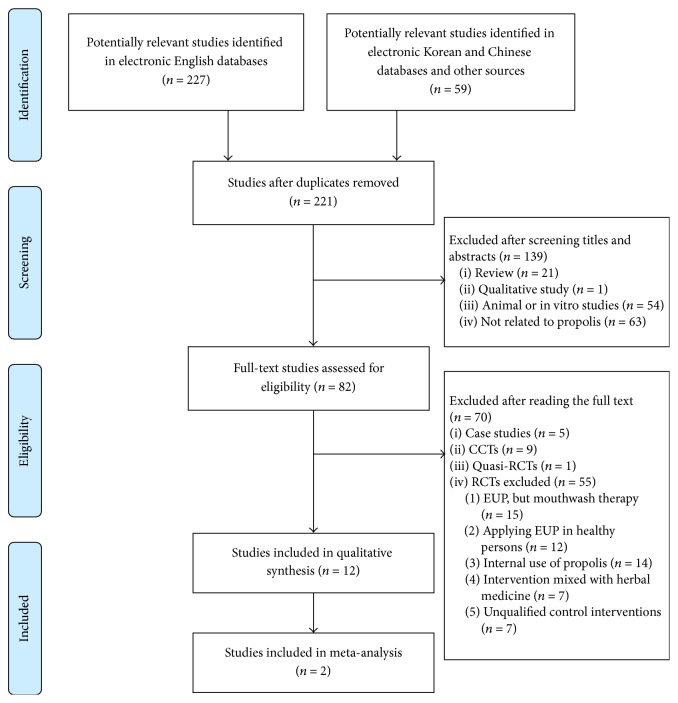
Flowchart of the RCT selection process. CCTs: controlled clinical trials; RCTs: randomized controlled trials; EUP: external use of propolis.

Of the 286 potentially relevant records, 221 studies were screened after duplicate trials were removed. Of these 221 studies, 139 were excluded because they were nonclinical trials (reviews, qualitative studies, and animal or in vitro studies) or were not related to propolis. Of the remaining 82 trials, 12 RCTs (English: *n* = 8; Chinese: *n* = 2; Korean: *n* = 1; Persian: *n* = 1) met our inclusion criteria (Figure 1).

Twelve studies were conducted in various countries, including two trials in Brazil and China and one trial in the Democratic Republic of Congo, Iran, Korea, Italy, Macedonia, Poland, Sudan and Ukraine each. We grouped the 12 trials into those addressing three diseases: five trials applied EUP for oral diseases (Table 1), five for skin diseases (Table 2), and two for genital diseases (Table 3).

### 3.2. Participants

#### 3.2.1. Number of Participants

The 12 studies included 862 participants. The sample size per group ranged from 10 to 52 participants. One study reported on 23 patients with two burn areas, one of which received EUP and the other a control intervention [24].

#### 3.2.2. Types of Disease

We classified the 12 RCTs into those addressing oral, skin, and genital diseases because the types of disease were heterogeneous. The five studies included in the oral diseases group consisted of three studies on recurrent oral aphthae (ROA) [20, 21, 23], one study on candidal stomatitis [21], and one on mucositis [23]. Five trials addressed skin diseases; second-degree burns [24], leg ulcers [25], tinea capitis and tinea versicolour [26], acne [27], and diabetes mellitus with foot ulcer [28] were each assessed by one study. Two studies were included in the genital diseases group; one was on acute vaginitis [29], and the other was on genital herpes [30].

### 3.3. Interventions

Four studies compared EUP with a placebo intervention that was the same form of EUP [19, 20, 30], and two studies compared EUP with EUP that had a different ingredient [19] and concentration [26]. A combination of EUP and other interventions was compared with a control of the same additional interventions in three trials [23, 25, 28]. Other studies compared EUP with mouthrinse [21], miconazole [21, 26], silver sulfadiazine [24], acacia honey [26], Vaseline [26], pine pollen mask packs [27], and metronidazole vaginal gel [28].

#### 3.3.1. Locations of Propolis Collected

A total of eight studies described the countries where propolis had been collected, including the UAE [19], Macedonia [20], Poland [25], Japan [26], Korea [27], China [28], Iran [29], and Canada [30].

#### 3.3.2. Chemical Composition of Propolis

The chemical composition of propolis was reported in only one study [20]. In this trial, the component of propolis was inhibitor against* Staphylococcus aureus*, minimum 62.5%, balm 55.0%, total phenols 24.2%, total flavones and flavonol 8.0%, and total flavonones and dihydroflavonols 49%.

#### 3.3.3. Types of EUP Form

Seven forms of EUP were used in 12 RCTs; extract and ointment were utilized in three trials each, and other types of EUP included paste [19], spray [20], gel [21], cream [24, 29], and mask pack [27].

#### 3.3.4. Amount of EUP Used

The amount of EUP used was reported in only four studies: the amount of EUP for 1 session ranged from 8 mg to 3 g in three trials [23, 27, 29], and one trial used 5 mL of EUP for 1 session [21].

### 3.4. Outcome Measures

Twelve studies reported on very diverse outcome measures due to the various types of diseases. The duration of treatment for each disease was investigated in three studies [19, 24, 25]. Two trials utilized Colony Forming Units (CFU), lesion size, and TER. Outcome measures related to pain were applied in three studies on oral diseases [19, 20, 22]. Studies on skin diseases used the measure of skin reactions such as stuff, papules, pustules, and pruritus [26, 27].

### 3.5. ROB Assessment

The included RCTs had a generally low methodological quality (Table 4). Although the 12 RCTs reported randomization, one study reported an inadequate method of random sequence generation (generated by even and odd numbers) [25], and the other 11 studies did not describe the method of randomization. Allocation was adequately concealed in only one study, in which it was managed by an external centre [23]. The participants and personnel were blinded in three trials (same form of intervention was used in the EUP and control groups) [20, 22, 27]. One study compared EUP with a placebo that was the same form of EUP, but it described a single-blinded method [19]. Blinding of the participants and outcome assessor was employed in one trial [21]. Nine studies properly addressed incomplete outcome data (dropout did not occur) [20–23, 25, 27–30]. The other three studies did not report the reasons for dropout [19, 24, 26]. Finally, for reporting bias, four trials reported their protocol before conducting the RCTs [21, 23, 26, 29].

### 3.6. Clinical Efficacy of EUP

A meta-analysis was not possible because of the heterogeneity in the diseases or outcome measures, with the exception of two trials on ROA [19, 22] that used a placebo for comparison. We summarized the results of the other studies because statistical pooling was not performed [20, 21, 23–30].

#### 3.6.1. Clinical Efficacy of EUP for Oral Diseases

**Figure 2:**
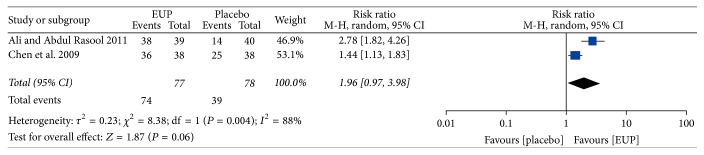
Meta-analysis of the total effective rate (TER) of EUP versus placebo. EUP: external use of propolis; CI: confidence intervals.

Of the five studies on oral diseases, a meta-analysis of two studies [19, 22] on ROA reported that there were no significant differences in TER for pain disappearance between EUP and placebo groups (RR = 1.96, 95% CI = 0.97–3.98, and *P* = 0.06) (Figure 2). For ROA, the effect of propolis spray was significantly better than placebo in measures of lesion size (*P* < 0.01 on the 8th day) and severity of pain (*P* < 0.01 on the 8th day) [20]. One trial [23] that compared propolis extract plus mouthrinse with mouthrinse alone showed a significant difference between groups using the National Cancer Institute-Common Terminology Criteria for Adverse Events (NCI-CTCAE) (*P* < 0.05). No significant difference between groups was identified in one trial [21].

#### 3.6.2. Clinical Efficacy of EUP for Skin Diseases

Of the five studies on skin disease, one on burns [24] showed no significant difference between groups in CFU or duration of treatment. For leg ulcers, the combination of propolis ointment with cointerventions was significantly more effective in reducing the duration of treatment (*P* < 0.01) than cointerventions alone in the control group [25]. For tinea capitis and tinea versicolour, propolis extract (50 mg/mL) showed a significant effect on pruritus, erythema, desquamation, and white blood cell count compared with Vaseline [26]. For acne, a pine pollen mask pack improved skin conditions and pustules better than a propolis mask pack [27]. One study [28] reported that propolis plus vasodilator therapy significantly improved the TER (*P* < 0.05) when compared with vasodilator therapy.

#### 3.6.3. Clinical Efficacy of EUP for Genital Diseases

Of the two studies on genital diseases, one study comparing propolis vaginal cream with metronidazole vaginal gel reported a significant improvement in Amsel's criteria and gram stain [29]. Another study [30] compared propolis ointment with acyclovir ointment and placebo and showed a significant effect of propolis on the number of healing patients (*P* < 0.01), crusted lesions (*P* < 0.001 on day 3), ulcer lesions (*P* < 0.05), vesicular lesions (*P* < 0.05), and herpetic-bacterial infections (*P* < 0.01); propolis ointment was significantly more effective at reducing ulcer lesions (*P* < 0.05) than placebo, but no significant difference was found between propolis and acyclovir in reducing ulcer lesions.

### 3.7. Adverse Events

Three studies described adverse events. One trial reported a skin reaction in 2 cases in the EUP group [23]. One patient suffered from itch in the control group [26]. No adverse events occurred in one study [30].

## 4. Discussion

The objective of this systematic review and meta-analysis was to provide evidence on EUP for any disease. A total of 12 studies were included in our review. Three studies on ROA [19, 20, 22] showed a significant effect of propolis treatment compared to the placebo groups. Two studies on skin diseases [25, 28] reported a significant effect of combined treatment of EUP with other interventions (e.g., rinsing therapy, compression treatment, or vasodilator therapy) compared to the other interventions alone. The results of one study on genital herpes [30] indicated a significant effect of 10 days of propolis ointment compared with a placebo ointment. We found in this systematic review that EUP has a more beneficial effect on ROA, skin diseases, and genital herpes than controls. However, because most of the RCTs had a small sample size, low methodological quality and groups receiving different forms of propolis, these analyses were not conclusive regarding the effectiveness of EUP for the studied diseases. Piredda et al. [23] reported that adverse events did not occur, and another trial [30] did not mention severe side effects with EUP. However, the evidence was insufficient to draw conclusions about the safety of EUP because only three studies [23, 26, 30] described adverse effects.

Most of the included trials had a low methodological quality based on Cochrane's risk of bias tool. Although the 12 RCTs stated that the participants were randomly assigned, an adequate method of randomization was not described, and only one [23] study had a low risk of bias for allocation concealment. Random sequence generation and allocation concealment are necessary to prevent selection bias. One out of 12 studies reported proper blinding in the outcomes assessment [21]. Low-quality blinding of outcome assessors is more likely to be influenced by placebo effects [33]. Therefore, the included trials have the potential for overestimation.

The propolis used in the 12 included RCTs was collected from diverse countries. Eight out of 12 studies [19, 20, 25–30] reported the locations of where the propolis was collected. Of the included twelve RCTs, only one study [20] mentioned chemical composition of propolis. Huang et al. [34] reported that propolis collected from many countries has similar chemical components but that there is a difference in concentration. Additionally, propolis collected from various regions in the same country has been identified as having a few distinct components [2]. The chemical composition of propolis collected from plants of certain countries can cause adverse effects [35]. Therefore, the efficacy and safety of propolis, considering geographical location, should be investigated in future studies.

Four studies used placebo interventions that used the same form of propolis for the control group [19, 20, 22, 30]; consideration of the smell of the placebo intervention was not mentioned. The use of an indistinguishable placebo compared to the experimental treatment is crucial for appropriate blinding of participants. Because propolis has a specific aromatic smell [36], patients who are familiar with propolis products may have been able to recognize whether the product was propolis. Thus, future studies should assess a proper placebo, considering the scent of propolis.

In this review, trials using propolis as a mouthwash were excluded because we focused on assessing the evidence of the efficacy of EUP when its ingredients were applied at the point of illness. There was some evidence in a recent dentistry study that propolis mouthwash protects against oral disease due to its antimicrobial properties [37]. One trial [21] compared propolis gel with propolis mouthwash and showed significant effects before and after treatment but no significant difference between groups. It is necessary to conduct comparative studies to identify the different efficacies of EUP between washing and applying forms of propolis.

Although our review indicated the applicability of propolis for external use, few studies on the standardization of EUP have been examined. Therefore, the following factors should be standardized to reduce the heterogeneity of future trials on EUP: (1) type of EUP form based on chemical composition of propolis; (2) effects and safety of EUP considering geographical locations; (3) amount used and number of treatment sessions; and (4) placebo model for EUP.

The strength of our review is that there was no restriction of language or publication status; hence, English, Chinese, Korean, and Persian papers were included in the review. However, there are some limitations to this systematic review. First, although 12 studies on EUP were included in this review, the heterogeneity in the diseases, the types of EUP form, control groups, and outcome measures was high; thus, a statistic pooling of 10 studies could not be assessed. In addition, it is difficult to propose any definitive conclusions regarding the safety of EUP because of the insufficient information on adverse effects. Therefore, researchers should conduct RCTs on EUP considering these limitations. Moreover, the side effects and amount used must be described in future studies to establish clinical practice guidelines for EUP.

As previous publications only investigated propolis for oral diseases [16], our review showed that propolis may be used externally for various diseases. The results could help determine the types of disease and forms of propolis for future research on EUP.

## 5. Conclusion

Our systematic review and meta-analysis suggested that the effectiveness of EUP for the treatment of oral, skin, and genital diseases was inconclusive because of the low methodological qualities and the small sample sizes. Further RCTs, with a high quality and large samples for specific disorders, must be conducted to provide additional clinical evidence on EUP treatment. Furthermore, the standardization of EUP to ensure clinical efficacy and safety is needed.

## Figures and Tables

**Table 1:** Characteristics of the included RCTs for oral diseases.

First author, year	Location of propolis production, chemical composition of propolis	Used form of propolis, amount used	Patient's disease, sample size (randomized/analysed)	Experimental group (intervention, regimen)	Control group (intervention, regimen)	Outcome measures	Main results	AE
Ali, 2011 [19]	UAE, n.r.	Paste, n.r.	Recurrent oral aphthae, 120/114	(A) PP (containing olive oil), *n* = 39, 2 times per day until healing	(B) PP (containing sesame oil), *n* = 35, 2 times per day until healing (C) Placebo paste, *n* = 40, 2 times per day until healing	(1) TER for pain disappearance (2) Duration of treatment for ulcer (3) Lesion size (4) Duration of drug adherence to mucous membrane	(1) (A)^a^, (B)^a^ significantly better than (C) (2) (A)^b^, (B)^b^ significantly better than (C) (3) (A) better than (B), (C) but NS (4) Most patients were approximately 20–30 minutes	n.r.

Atanasovska, 2014 [20]	Macedonia, I 62.5% + B 55% + TP 24.2% + TFF 8% + TFD 49%	Spray, n.r.	Recurrent oral aphthae, 20/20	(A) PS (Proaftol), *n* = 10, 24–32 sessions (3-4 times per day for 8 days)	(B) Placebo spray, *n* = 10, 24–32 sessions (3-4 times per day for 8 days)	(1) Lesion size (2) Severity of pain	(1) On day 3, Positive^c^; on day 5, Positive^c^; on day 8, Positive^b^ (2) On day 3, Positive^c^; on day 5,Positive^b^; on day 8, Positive^b^	n.r.

Capistrano, 2013 [21]	n.r., n.r.	Gel, 1 session 5 mL	Candidal stomatitis, 45/45	(A) PG, *n* = 15, 64 sessions (4 times per day for 14 days)	(B) Mouthrinse (containing propolis), *n* = 15, 64 sessions (4 times per day for 14 days) (C) Miconazole, *n* = 15, 64 sessions (4 times per day for 14 days)	(1) CFU (2) Newton's classification	(1) Significant difference in (A)^c^, (B)^c^, (C)^c^ but NS in each group (2) Significant difference in (A)^b^, (B)^c^, (C)^b^ but NS in each group	n.r.

Chen, 2009 [22]	n.r., n.r.	Extract, n.r.	Recurrent oral aphthae, 76/76	(A) PE, *n* = 38, 14 sessions (2 times per day for 7 days)	(B) Placebo (oral antiseptics), *n* = 38, 14 sessions (2 times per day for 7 days)	(1) TER for ROA (2) TER for pain disappearance	(1) Positive^b^ (2) Positive^b^	n.r.

Piredda, 2015 [23]	n.r., n.r.	Extract, 1 session 8–10 mg	Oral mucositis, 60/60	(A) PE + mouthrinse, *n* = 30, 15 sessions (1 time per day for 15 days)	(B) Mouthrinse, *n* = 30, 15 sessions (1 time per day for 15 days)	(1) NCI-CTCAE version 4.0	(1) Positive^a^	Manifested suspected skin reaction (2 in group (A))

^a^ *P* < 0.05; ^b^*P* < 0.01; ^c^*P* < 0.001.

AE: adverse events; B: balm (extract with 70% ethanol); CFU: colony forming units; I: inhibitor against *Staphylococcus aureus*; NCI-CTCAE: National Cancer Institute-Common Terminology Criteria for Adverse Events; n.r.: not reported; NS: no significant difference between groups; PE: propolis extract; PG: propolis gel; positive: (A) significantly better than (B); PP: propolis paste; PS: propolis spray; ROA: recurrent oral aphthae; TER: total effective rate; TFD: total flavonones and dihydroflavonols; TFF: total flavones and flavonol; TP: total phenols.

**Table 2:** Characteristics of the included RCTs for skin diseases.

First author, year	Location of propolis production, chemical composition of propolis	Used form of propolis, amount used	Patient's disease, sample size (randomized/analysed)	Experimental group (intervention, regimen)	Control group (intervention, regimen)	Outcome measures	Main results	AE
Gregroy, 2002 [24]	n.r., n.r.	Cream, n.r.	Second-degree burns, 33/23^d^	(A) PC, *n* = 23^d^, 1 session	(B) SSD, *n* = 23^d^, 1 session	(1) CFU (2) Duration of treatment for burns	(1) NS (2) (A) better than (B) but NS	n.r.

Kucharzewski, 2013 [25]	Poland, n.r.	Ointment, n.r.	Varicose veins of lower extremities with ulcer, 56/56	(A) PO + rinsing the ulcer with PSCS + compression treatment, *n* = 28, PO: 7 to 42 sessions (1 time per day until healing); the others: 7 to 42 sessions (1 time per week until healing)	(B) Rinsing the ulcer with PSCS + compression treatment, *n* = 28, 28 to 102 sessions (1 time per week until healing)	(1) Duration of treatment for ulcer	(1) Positive^b^	n.r.

Ngatu, 2011 [26]	Japan, n.r.	Extract, n.r.	Tinea capitis and tinea versicolour, 242/188	(A) PE (50 mg/mL), *n* = 26, 28 sessions (1 time per day for 28 days)	(B) PE (100 mg/mL), *n* = 29, 28 sessions (1 time per day for 28 days) (C) Acacia honey, *n* = 31, 28 sessions (1 time per day for 28 days) (D) Miconazole, *n* = 50, 28 sessions (1 time per day for 28 days) (E) Vaseline, *n* = 52, 28 sessions (1 time per day for 28 days)	(1) Pruritus (2) Papule, pustule (3) Erythema (4) Desquamation (5) WBC (6) Leukocytes	(1) (A)^b^, (B)^b^, (C)^a^, (D)^b^ significantly better than (E) (2) Significant difference in (A)^b^, (B)^b^, (C)^b^, (D)^c^ (3) (A)^c^, (B)^c^, (C)^c^, (D)^c^ significantly better than (E) (4) (A)^c^, (B)^c^, (C)^c^, (D)^c^ significantly better than (E) (5) (A)^b^, (B)^a^, (D)^a^, significantly better than (E), but NS between (C) and (E) (6) NS	Itch (1 in group (D))

Park, 2013 [27]	Korea, n.r.	Mask pack, 2 g	Acne, 30/30	(A) PMP, *n* = 15, 10 sessions (1 time per week for 4 weeks)	(B) PPMP, *n* = 15, 10 sessions (1 time per week for 4 weeks)	(1) Skin conditions ① Moisture ② Sebum ③ Elasticity (2) Number of acne lesions ① Stuff ② Papule ③ Pustule	(1) ① Negative^b^ ②, ③ Negative^a^ (2) ①, ② NS ③ Negative^a^	n.r.

Yin, 2013 [28]	China, n.r.	Ointment, n.r.	Other specified diabetes mellitus with foot ulcer, 60/60	(A) PO + VT, *n* = 30, PO: 4 times per day for 17 months, VT: 24 months	(B) VT, *n* = 30, 24 months	(1) TER for ulcer	(1) Positive^a^	n.r.

^a^ *P* < 0.05; ^b^*P* < 0.01; ^c^*P* < 0.001; ^d^same patients with two burn areas received PC and SSD.

AE: adverse events; CFU: colony forming units; negative: (B) significantly better than (A); n.r.: not reported; NS: no significant difference between groups; PC: propolis cream; PE: propolis extract; PMP: propolis mask pack; PO: propolis ointment; positive: (A) significantly better than (B); PPMP: pine pollen mask pack; PSCS: physiological sodium chloride solution; SSD: silver sulfadiazine; TER: total effective rate; VT: vasodilator therapy; WBC: white blood cell.

**Table 3:** Characteristics of the included RCTs for genital diseases.

First author, year	Location of propolis production, chemical composition of propolis	Used form of propolis, amount used	Patient's disease, sample size (randomized/analysed)	Experimental group (intervention, regimen)	Control group (intervention, regimen)	Outcome measures	Main results	AE
Mousavi, 2016 [29]	Iran, n.r.	Cream, 1 session 3 g	Acute vaginitis, 100/100	(A) PVC, *n* = 50, 7 sessions (1 time per day for 7 days)	(B) MVG, *n* = 50, 7 sessions (1 time per day for 7 days)	(1) Amsel's criteria (2) Gram stain	(1) Positive^a^ (2) Positive^a^	n.r.

Vynograd, 2000 [30]	Canada, n.r.	Ointment, n.r.	Herpes viral infection of genitalia and urogenital tract, 90/90	(A) PO, *n* = 30, 40 sessions (4 times per day for 10 days)	(B) AO, *n* = 30, 40 sessions (4 times per day for 10 days) (C) Placebo ointment, *n* = 30, 40 sessions (4 times per day for 10 days)	(1) Number of patients who were healed (2) Lesions ① Crusted lesions ② Ulcer lesions ③ Vesicular lesions (3) Infection of herpetic-bacterial (only for 28 infected women)	(1) Positive^b^ (2) ① On day 3, Positive^c^ ② (A) better than (B) but NS; (A)^a^ significantly better than (C) ③ Positive^a^ (3) Positive^b^	None

^a^ *P* < 0.05; ^b^*P* < 0.01; ^c^*P* < 0.001.

AE: adverse events; AO: acyclovir ointment; MVG: metronidazole vaginal gel; n.r.: not reported; PO: propolis ointment; positive: (A) significantly better than (B); PVC: propolis vaginal cream.

**Table 4:** Risk of bias assessment.

First author, year	Selection bias		Performance bias	Detection bias	Attrition bias	Reporting bias
	Random sequence generation	Allocation concealment	Blinding of participants and personnel	Blinding of outcome assessment	Incomplete outcome data	Selective reporting
Ali, 2011 [19]	U	U	H	U	U	U
Atanasovska, 2014 [20]	U	U	L	U	L	U
Capistrano, 2013 [21]	U	U	H	L	L	L
Chen, 2009 [22]	U	U	L	U	L	U
Piredda, 2015 [23]	U	L	H	U	L	L
Gregroy, 2002 [24]	U	U	H	U	U	U
Kucharzewski, 2013 [25]	H	U	H	U	L	U
Ngatu, 2011 [26]	U	U	H	U	U	L
Park, 2013 [27]	U	U	L	U	L	U
Yin, 2013 [28]	U	U	H	U	L	U
Mousavi, 2016 [29]	U	U	H	U	L	L
Vynograd, 2000 [30]	U	U	H	U	L	U

H: high risk; L: low risk; U: unclear risk.
